# Treatment Equity in the Immunotherapy Era: Options for Patients with Both Autoimmune Disease and GU Cancers

**DOI:** 10.3390/life12030360

**Published:** 2022-03-02

**Authors:** Gavin Hui, Claire Drolen, Christopher A. Hannigan, Alexandra Drakaki

**Affiliations:** 1 Department of Medicine, Stanford University, Stanford, CA 94305, USA; gavinhui@stanford.edu; 2 Department of Medicine, David Geffen School of Medicine, UCLA, Los Angeles, CA 90095, USA; cdrolen@mednet.ucla.edu; 3 Department of Medicine, Division of Hematology/Oncology, David Geffen School of Medicine, UCLA, Los Angeles, CA 90095, USA; channigan@mednet.ucla.edu

**Keywords:** immune checkpoint inhibitors, autoimmune disease, renal cancer, bladder cancer, immune-related adverse event, immunotherapy

## Abstract

Numerous immunotherapeutic agents, such as immune checkpoint inhibitors (ICIs), have been approved for the treatment of genitourinary (GU) malignancies. While ICIs have improved treatment outcomes and expanded treatment options, they can cause immune-related adverse events (irAEs). The scope of irAEs is broad, and this paper aims to review the rheumatologic side effects associated with immunotherapy drugs approved for bladder cancer and renal cell carcinoma. IrAEs are graded by the common terminology criteria for adverse events (CTCAE), which ranges from 1 to 5. The management of irAEs includes corticosteroids or other immunosuppressive therapies, and it may require discontinuation of immunotherapy. Several real world experience studies suggest that most patients with pre-existing autoimmune diseases treated with ICI did not have to discontinue treatment due to immune-mediated side effects. While data suggest autoimmune side effects are manageable, patients with pre-existing autoimmune diseases are often excluded from immunotherapy clinical trials. Better understanding of these irAEs will improve its safety and expand its use in those with underlying autoimmune disease.

## 1. Introduction

Genitourinary malignancies include primarily the cancers of the prostate, bladder and kidneys, and testicular and penile cancers occur less frequently. In 2021, the US had an estimated 83,730 new cases and 17,200 deaths from bladder cancer [1]. In addition, the US had an estimated 76,080 new cases and an estimated 13,780 deaths from kidney cancer in 2021 [2].

While GU malignancies are prevalent, recent discoveries in cancer biology and therapeutics, particularly the addition of immunotherapeutic agents, suggest that the tide may be turning for the treatment of these diseases. The most established application of cancer immunotherapy is immune checkpoint inhibitors (ICIs). A class of monoclonal antibodies, ICIs, act to enhance T-cell antitumor immune surveillance through action on three key targets: programmed cell death receptor 1 (PD-1), programmed cell death ligand 1 (PD-L1), and cytotoxic T lymphocyte-associated antigen 4 (CTLA-4). Numerous drugs within the ICI class have been approved for the treatment of kidney, bladder cancer, and other non-GU cancers. These include the PD-1 inhibitors nivolumab and pembrolizumab, the PD-L1 inhibitor avelumab, and combination therapies have shown benefits in overall and progression free survival [3,4,5]. These important benefits of ICI therapy are balanced by a significant risk of “off target” adverse effects, formally known as immune-related adverse events (irAEs), which occur as a result of unregulated immune activity against non-cancer tissue. One recent study estimates that irAEs will affect nearly 60% of patients treated with combination ipilimumab and nivolumab [6]. The scope of irAEs is also quite broad, with dozens of distinct irAEs affecting nearly every organ system described in the literature [7]. While significant attention has been directed towards characterizing and quantifying the scope of irAEs, comparatively little is known of the rheumatologic side effects of ICIs and the effects of ICIs on patients with pre-existing autoimmune or rheumatologic disease.

Autoimmune diseases such as rheumatoid arthritis (RA), inflammatory bowel disease (IBD), systemic lupus erythematosus (SLE), and multiple sclerosis (MS), among many others, are highly prevalent, affecting somewhere between 24 and 50 million people in North America alone. RA prevalence has been estimated to be around 0.5–1% in the US and northern European countries, and RA incidence is estimated to be 40 per 100,000 persons annually [8]. In addition, the prevalence of IBD in North America was above 0.3% [9]. There is evidence of an association between autoimmune disease and increased risk of cancer, including bladder and kidney cancer [10,11]. Despite this association, patients with pre-existing autoimmune disease have often been excluded from clinical trials of ICIs based on concern for exacerbating or triggering further autoimmunity.

In this review, we aim to characterize the spectrum of immune-mediated side effects reported for the immunotherapies used in treating GU malignancies, primarily bladder and kidney cancer. In addition, we focus on treatment side effects, both as de novo and as potential flare of pre-existing autoimmune symptoms.

## 2. Immunotherapy Drugs in Bladder Cancer

Several ICI drugs have been approved for advanced bladder cancer (Table 1). In particular, anti PD-1 and PD-L1 therapies have been used in the second-line for patients who have progressed during or after platinum-based chemotherapy. Avelumab, nivolumab, and pembrolizumab have been approved as second-line therapy [3,4,12]. Durvalumab and atezolizumab were given accelerated approval for similar indications, but they were recently withdrawn given new study results showing no OS benefit over chemotherapy [5,13].

While the preferred treatment option for treatment-naïve patients with advanced bladder cancer is platinum-based chemotherapy, pembrolizumab and atezolizumab can also be offered in the first-line setting for patients who are cisplatin ineligible and have PD-L1 positive expressing tumors or who are platinum ineligible [4,14].

## 3. Immunotherapy Drugs in RCC

Pembrolizumab, nivolumab, avelumab, and ipilimumab are all approved for treatment of mRCC in the first and second-line settings. Treatment selection depends on the evaluation of the patients’ risk profile and stratification. Risk is determined based on the International Metastatic Renal Cell Carcinoma Database Consortium (IMDC) criteria [15,16]. In addition, Motzer et al. have developed a five factor prognostic model that stratifies patients into low, intermediate, and high risk groups [17]. It is worth noting, however, that IMDC and Motzer risk stratification algorithms were developed and reported before the use of immunotherapy in frontline mRCC treatment.

Many of the regimens used to treat mRCC combine ICI agents with vascular endothelial growth factor (VEGF) inhibitors, which have been the standard-of-care treatments for mRCC since their marketing approvals in the mid-2000s. The VEGF inhibitors approved for the treatment of mRCC are sunitinib, pazopanib, cabozantinib, axitinib, sorafenib, lenvatinib, and tivozanib [18,19,20,21].

For patients that have been determined to have favorable risk disease, combinations of pembrolizumab plus axitinib, pembrolizumab plus lenvatinib, nivolumab plus cabozantanib, nivolumab plus ipilimumab, and avelumab plus axitinib are approved as first-line treatment options [22,23,24,25,26,27,28,29].

Patients with intermediate or high risk disease have similar treatment options when it comes to immunotherapy. Combinations of pembrolizumab plus lenvatinib or pembrolizumab plus axitinib or nivolumab plus cabozantinib or nivolumab plus ipilimumab are all approved treatment strategies in this risk class. Avelumab plus axitinib combination therapy has also recently been approved. Among these options, clinical trials with pembrolizumab plus axitinib and nivolumab plus cabozantinib have demonstrated the most significant treatment benefit for patients with non-favorable risk [22,25,29].

## 4. Treatment-Related Immune-Mediated Side Effects

IrAEs are categorized clinically via the common terminology criteria for adverse events (CTCAE) [30]. CTCAE grading ranges from 1 to 5, and lower grades indicate more mild adverse events that can be managed usually with supportive measures alone, are reversible, and patients can remain on treatment. At the other end of the spectrum, grade 5 is usually a fatal toxicity. Commonly reported immune-mediated events across the ICIs discussed above can be categorized into systemic effects, dermatitis, enterocolitis, endocrinopathies, and arthritis [31]. Each side effect presents at varying frequency and severity depending on the specific ICI, combination with other anti-tumor agents, length of treatment, and treatment population. The common symptoms are fatigue, pruritus, rash, diarrhea, and joint pain.

In patients with locally advanced or metastatic urothelial carcinoma on ICI monotherapies, Grade 1 or 2 diarrhea per CTCAE is seen in 7% on nivolumab, 8.4% on durvalumab, 9.0% on pembrolizumab, 16.6% on avelumab, and 12% on atezolizumab [3,4,13,14]. In the same patient population, these rates significantly increased in combination therapies where Grade 1 or 2 diarrhea was seen in 23.1% and 32.6% on nivolumab and ipilimumab. However, these therapies provided superior efficacy [3].

The frequency of Grade 1 or 2 events differs significantly in metastatic renal cell carcinoma (mRCC) patients on monotherapy with 18% seen on nivolumab, 13.9% on pembrolizumab, 12.9% on avelumab, and 11% on atezolizumab [32,33,34,35]. Conversely, rates are nearly equal for those on ICI combination regimens with 27% on nivolumab and ipilimumab [26].

Some serious but rare irAEs, such as pneumonitis, occur with both anti CTLA-4 and anti PD-1 agents in a small minority of patients but can quickly become life-threatening. The combination of these therapies increases the likelihood, demonstrated by the 2% incidence on nivolumab and ipilimumab compared with <1% on nivolumab, 1% on ipilimumab, and 1% on pembrolizumab [3,36,37]. Pulmonary co-morbidities are significant indicators of this event.

## 5. Mechanism and Clinical Presentation of Autoimmunity

ICIs can have off-target tissue effects due to non-specific T cell activation from blocking inhibitory pathways. PD-1 and PD-L1 are both widely expressed in different cells, such as T cells and B cells. The interaction between PD-1 and its ligands blocks T-cell response such as cytokine release and cell proliferation. Therefore, blocking this interaction leads to the release of activated immune responses [38]. While this leads to a greater immune reaction against the cancer, the non-specific nature of this activation can cause immune-related adverse effects (irAEs).

Several publications have tried to characterize the frequency of these irAEs. For example, a large tertiary cancer center in Israel found rheumatic manifestations in 14 of 400 patients (3.5%) [39]. The most common manifestation observed was inflammatory arthritis (85%). This is consistent with other reports that inflammatory arthritis with joint pain, swelling, and tenderness is one of the most commonly reported irAEs [40,41]. These irAEs have been seen even in patients without prior rheumatologic conditions. This is supported by a case series from a rheumatology department describing 13 patients without pre-existing autoimmune diseases developing irAEs. These irAEs included arthritis, sicca syndrome, polymyalgia rheumatica, and inflammatory myositis [42].

While many irAEs are characterized to be inflammatory arthritis, RA becomes an important differential. Rheumatoid arthritis (RA) is a polyarthritis, with most cases characterized by seropositivity in rheumatoid factor (RF) and/or anti-cyclic citrullinated peptide (anti-CCP). Most patients with inflammatory arthritis after ICI therapy have been seronegative. However, there is a report of de novo RA developing in patients receiving ICI therapy. These patients did not have pre-existing rheumatologic disease. Out of 10 patients, 6 (60%) of them were positive for anti-CCP antibodies and 4 (40%) were positive for RF [43].

Polymyalgia rheumatica (PMR) is a condition with proximal muscle stiffness and pain without weakness. It can sometimes occur with giant cell arteritis (GCA). This condition has also been described in patients treated with ICIs [42]. One meta-analysis of 49 patients with reported PMR-like side effects from ICI found that about 28 (75%) of the cases fulfilled complete criteria for PMR [44].

Myopathy is also a frequently reported side effect of those on ICI. Two inflammatory myopathies, dermatomyositis and polymyositis, have been described as irAEs [45]. These two conditions are characterized by proximal muscle weakness and muscle inflammation. Dermatomyositis has additional skin manifestations compared to polymyositis. While there is a case report of myositis associated with nivolumab, other reports of myositis have been noted in CTLA-4 blockade, which is not a drug used for bladder cancer [45,46]. Other rarer cases of irAEs have been reported, such as scleroderma in a few patients receiving pembrolizumab [47].

## 6. Pre-Existing Rheumatologic Disease

Patients with pre-existing autoimmune diseases have been excluded from immunotherapy based clinical trials, and pre-existing autoimmune diseases have been identified in a meta-analysis to be a risk factor for irAE incidence. As one may expect, based on several prospective and retrospective studies, there were higher irAEs in those with pre-existing autoimmune diseases compared to those without [48,49,50]. Table 2 includes several studies of patients with pre-existing autoimmune disease who had treatment with ICI.

A larger systemic meta-analysis characterized irAEs in patients with pre-existing autoimmune diseases. The incidence of pre-existing autoimmune disease flare was 35% and incidence of irAEs was 33%. This study illustrated that immune toxicities are common in patients with pre-existing autoimmune diseases treated with ICIs. The patients with RA in this study also had a higher flare occurrence compared to those with other pre-existing autoimmune conditions (RR 1.25–1.88) [51]. RA flares tend to be common in these cohorts. Another retrospective cohort study included 112 patients who had pre-existing autoimmune diseases, and the most frequent diagnoses in this study were psoriasis, RA, and IBD. This study had one of the highest incidences, with 71% of these patients developing autoimmune disease flare and/or other irAEs [48,52].

Specifically in the setting of locally advanced or metastatic urothelial carcinoma, a study of atezolizumab treatment of 997 patients showed that 35 patients had pre-existing autoimmune diseases. Psoriasis was in 15 out of 35 of these patients, and there were more adverse events in the 35 patients with pre-existing autoimmune diseases [8]. However, even with increased adverse events, the was no increase in treatment-related deaths [8]. Another multicenter retrospective study looked at patients with advanced RCC and urothelial cancer (UC) with pre-existing autoimmune disease. In total, 106 patients, 58 RCC and 48 UC, were included in this study. A total of 38 (36%) patients experienced a flare, and 40 (38%) patients were found to have new irAEs [53].

Given that patients with pre-existing autoimmune diseases were not participating in clinical trials, real world data has been helpful to characterize the side effects of ICIs for those with pre-existing autoimmune disease. While data suggests that patients with a history of autoimmune diseases do experience flares with ICI treatment, the management and incidences of more severe irAEs do not seem to be significantly higher compared to those without pre-existing autoimmune conditions [54].

## 7. Management for Patients with Cancer and irAEs

IrAEs associated with ICIs can be treated with corticosteroids in most cases or tumor necrosis factor-α (TNFα) inhibitors in certain toxicities such as colitis. Immunosuppressive therapy with mycophenolate mofetil is recommended in patients with steroid refractory immune-mediated hepatitis, while hormonal replacement is indicated in certain endocrinopathies. Anti-interleukin (IL)-6 receptor antibody has been approved for the treatment of RA. There is a case series of three patients with arthritis while undergoing ICI therapy. They were treated with tocilizumab, an approved anti IL-6 agent, and demonstrated clinical improvement [55]. Furthermore, there have been case reports of patients with immune-mediated myocarditis treated with CTLA-4 agonist [56].

ICIs can frequently induce new irAEs as well as flares of existing autoimmune diseases. Studies seem to suggest that most patients experiencing flares or irAEs of an autoimmune syndrome can be mostly managed without discontinuation of ICI [9,50]. In addition, other observational studies with 524 patients found that about 6.6% of patients receiving ICIs were referred to rheumatology for irAEs. Almost all these patients were receiving anti PD-1/PD-L1 antibody treatment. It was encouraging that all of the patients in this study did not have to discontinue ICI because they responded to steroids or symptomatic treatments [40].

In a cohort of 112 patients with pre-existing autoimmune diseases treated with ICI, only about 21% of them needed permanent discontinuation of ICI, while less than half of them needed immunosuppressive therapy. While this study highlighted the adverse effects of ICIs in those with autoimmune disease, it provided real world evidence to support the notion that most of these irAEs can be managed without discontinuing ICI [52].

Other studies involving patients with locally advanced or metastatic urothelial carcinoma receiving atezolizumab showed no significant difference in treatment-related deaths or in ICI discontinuation [8]. The ICI efficacy, as suggested by the median OS, did not differ between those with and without pre-existing autoimmune diseases in this study. Another study with advanced RCC and urothelial cancer (UC) with pre-existing autoimmune disease, 17 (45%) patients with a flare required steroids and 6 (16%) required discontinuing ICI [53]. Overall, the toxicities of ICI therapy were manageable. Outside of UC and RCC, there is also real world evidence that ICI therapy in those with pre-existing autoimmune conditions infrequently requires discontinuation due to irAEs [49,57].

While data outside of clinical trials suggest that autoimmune side effects are manageable, even in those with pre-existing autoimmune disease, there are currently no clinical trial results. However, there is a phase 1B trial underway to study the side effects of nivolumab and how it works in patients with autoimmune disease and metastatic or unresectable cancers. The primary objective of the study is to assess overall safety with the use of nivolumab. Autoimmune disorders included in this study include dermatomyositis, systemic sclerosis, RA, SLE, IBD, MS and Sjogren’s syndrome. This study is ongoing and is projected to be completed in August 2022 [58].

## 8. Conclusions

There is growing evidence that patients who develop irAEs have an increased chance of responding to therapy while having an underlying autoimmune condition should not be a limiting factor for treating a potentially terminal disease [59]. We hope, therefore, that with a better understanding of the various irAEs, the next generation of studies will combine ICIs and immunomodulating agents with the goal of a prolonged quality and quantity of life for those suffering from cancer with or without underlying autoimmune disease.

## Figures and Tables

**Table 1:** Immunotherapy approved for metastatic urothelial carcinoma (mUC).

Drug	Mechanism	Trial	Indications
Pembrolizumab	Anti PD-1	KEYNOTE-045 (second-line) KEYNOTE-052 (first-line)	Second-line: progression during or following platinum-based chemotherapy First-line: not eligible for platinum-based chemotherapy
Nivolumab	Anti PD-1	CheckMate-275	Second-line: progression during or following platinum-based chemotherapy
Avelumab	Anti PD-L1	JAVELIN	Second-line: progression during or following platinum-based chemotherapy Maintenance therapy after first line platinum-based chemotherapy
Atezolizumab	Anti PD-L1	IMvigor210	First-line: not eligible for cisplatin-based chemotherapy and tumors express PD-L1 ≥5% OR not eligible for any platinum-based therapy

**Table 2:** Studies of ICI in patients with advanced malignancy and pre-existing autoimmune disease.

Study	Study Design	Cohort Size, *n*	Female Sex, *n* (%)	Cohort Geography	Cancer Sites (*n*)	ICI Used (*n*)	Incidence of AD Flare, *n* (%)	Incidence of New irAE, *n* (%)	ICI Permanently Discontinued, *n* (%)
Danlos 2018	Single center, prospective	45	24 (53.3)	France	Skin (36), lung (6), other (3)	Pembrolizumab (34), nivolumab (10), avelumab (1)	11 (24.4)	10 (22.2)	5 (11.1)
Alexander 2020	Single center, retrospective	42	18 (42.8)	USA	Skin (25), lung (11), GU (4), other (2)	Pembrolizumab (23), nivolumab (13), ipilimumab (5), atezolizumab (1)	12 (28.6)	9 (21.4)	9 (21.4)
Fountzilas 2021	Multicenter, retrospective	123	46 (37.4)	Greece	Lung (84), skin (18), GU (7), head and neck (6), other (8)	Pembrolizumab (50), nivolumab (51), atezolizumab (8), durvalumab (5), ipilimumab (4), avelumab (1), ipilimumab + nivolumab (3)	31 (25.2)	43 (35.0)	11 (8.9%)
Martinez Chanza 2020	Multicenter, retrospective	106	31 (29.2)	USA and EU	Kidney (57), bladder (48)	Not specified	38 (35.8)	40 (37.7)	7 (6.6)

## Data Availability

Not applicable.

## References

[1] Key Statistics for Bladder Cancer. https://www.cancer.org/cancer/bladder-cancer/about/key-statistics.html.

[2] Key Statistics About Kidney Cancer. https://www.cancer.org/cancer/kidney-cancer/about/key-statistics.html.

[3] Sharma P., Retz M., Siefker-Radtke A., Baron A., Necchi A., Bedke J., Plimack E.R., Vaena D., Grimm M.-O., Bracarda S. (2017). Nivolumab in metastatic urothelial carcinoma after platinum therapy (CheckMate 275): A multicentre, single-arm, phase 2 trial. Lancet Oncol..

[4] Fradet Y., Bellmunt J., Vaughn D.J., Lee J.L., Fong L., Vogelzang N.J., Climent M.A., Petrylak D.P., Choueiri T.K., Necchi A. (2019). Randomized phase III KEYNOTE-045 trial of pembrolizumab versus paclitaxel, docetaxel, or vinflunine in recurrent advanced urothelial cancer: Results of >2 years of follow-up. Ann. Oncol..

[5] Patel M.R., Ellerton J., Infante J.R., Agrawal M., Gordon M., Aljumaily R., Britten C.D., Dirix L., Lee K.-W., Taylor M. (2017). Avelumab in metastatic urothelial carcinoma after platinum failure (JAVELIN Solid Tumor): Pooled results from two expansion cohorts of an open-label, phase 1 trial. Lancet Oncol..

[6] Larkin J., Chiarion-Sileni V., Gonzalez R., Grob J.-J., Rutkowski P., Lao C.D., Cowey C.L., Schadendorf D., Wagstaff J., Dummer R. (2019). Five-Year Survival with Combined Nivolumab and Ipilimumab in Advanced Melanoma. N. Engl. J. Med..

[7] Ramos-Casals M., Brahmer J.R., Callahan M.K., Flores-Chávez A., Keegan N., Khamashta M.A., Lambotte O., Mariette X., Prat A., Suárez-Almazor M.E. (2020). Immune-related adverse events of checkpoint inhibitors. Nat. Rev. Dis. Prim..

[8] Hunter T.M., Boytsov N.N., Zhang X., Schroeder K., Michaud K., Araujo A.B. (2017). Prevalence of rheumatoid arthritis in the United States adult population in healthcare claims databases, 2004–2014. Rheumatol. Int..

[9] Worldwide Incidence and Prevalence of Inflammatory Bowel Disease in the 21st Century: A Systematic Review of Population-Based Studies—PubMed. https://pubmed.ncbi.nlm.nih.gov/29050646/.

[10] El-Refai S.M., Brown J.D., Black E.P., Talbert J. (2017). Immune Checkpoint Inhibition and the Prevalence of Autoimmune Disorders Among Patients with Lung and Renal Cancer. Cancer Inform..

[11] Liu X., Ji J., Forsti A., Sundquist K., Sundquist J., Hemminki K. (2013). Autoimmune Disease and Subsequent Urological Cancer. J. Urol..

[12] Atezolizumab in Patients with Locally Advanced and Metastatic Urothelial Carcinoma Who Have Progressed Following Treatment with Platinum-Based Chemotherapy: A Single-Arm, Multicentre, Phase 2 Trial—PubMed. https://pubmed.ncbi.nlm.nih.gov/26952546/.

[13] Powles T., O’Donnell P.H., Massard C., Arkenau H.-T., Friedlander T.W., Hoimes C., Lee J.L., Ong M., Sridhar S.S., Vogelzang N.J. (2017). Efficacy and Safety of Durvalumab in Locally Advanced or Metastatic Urothelial Carcinoma. JAMA Oncol..

[14] Balar A.V., Galsky M.D., Rosenberg J.E., Powles T., Petrylak D.P., Bellmunt J., Loriot Y., Necchi A., Hoffman-Censits J., Perez-Gracia J.L. (2017). Atezolizumab as first-line treatment in cisplatin-ineligible patients with locally advanced and metastatic urothelial carcinoma: A single-arm, multicentre, phase 2 trial. Lancet.

[15] Heng D.Y., Xie W., Regan M.M., Warren M.A., Golshayan A.R., Sahi C., Eigl B., Ruether J.D., Cheng T., North S. (2009). Prognostic Factors for Overall Survival in Patients with Metastatic Renal Cell Carcinoma Treated with Vascular Endothelial Growth Factor–Targeted Agents: Results from a Large, Multicenter Study. J. Clin. Oncol..

[16] Heng D.Y., Xie W., Regan M.M., Harshman L.C., Bjarnason G.A., Vaishampayan U.N., Mackenzie M., Wood L., Donskov F., Tan M.-H. (2013). External validation and comparison with other models of the International Metastatic Renal-Cell Carcinoma Database Consortium prognostic model: A population-based study. Lancet Oncol..

[17] Motzer R.J., Mazumdar M., Bacik J., Berg W., Amsterdam A., Ferrara J. (1999). Survival and Prognostic Stratification of 670 Patients With Advanced Renal Cell Carcinoma. J. Clin. Oncol..

[18] Motzer R.J., Hutson T.E., Tomczak P., Michaelson M.D., Bukowski R.M., Rixe O., Oudard S., Negrier S., Szczylik C., Kim S.T. (2007). Sunitinib versus Interferon Alfa in Metastatic Renal-Cell Carcinoma. N. Engl. J. Med..

[19] Choueiri T.K., Escudier B., Powles T., Mainwaring P.N., Rini B.I., Donskov F., Hammers H., Hutson T.E., Lee J.-L., Peltola K. (2015). Cabozantinib versus Everolimus in Advanced Renal-Cell Carcinoma. N. Engl. J. Med..

[20] Choueiri T.K., Escudier B., Powles T., Tannir N.M., Mainwaring P.N., Rini B.I., Hammers H.J., Donskov F., Roth B.J., Peltola K. (2016). Cabozantinib versus everolimus in advanced renal cell carcinoma (METEOR): Final results from a randomised, open-label, phase 3 trial. Lancet Oncol..

[21] Sternberg C.N., Davis I.D., Mardiak J., Szczylik C., Lee E., Wagstaff J., Barrios C.H., Salman P., Gladkov O.A., Kavina A. (2010). Pazopanib in Locally Advanced or Metastatic Renal Cell Carcinoma: Results of a Randomized Phase III Trial. J. Clin. Oncol..

[22] Rini B.I., Plimack E.R., Stus V., Gafanov R., Hawkins R., Nosov D., Pouliot F., Alekseev B., Soulières D., Melichar B. (2019). Pembrolizumab plus Axitinib versus Sunitinib for Advanced Renal-Cell Carcinoma. N. Engl. J. Med..

[23] Powles T., van der Heijden M.S., Castellano D., Galsky M.D., Loriot Y., Petrylak D.P., Ogawa O., Park S.H., Lee J.-L., De Giorgi U. (2020). Durvalumab alone and durvalumab plus tremelimumab versus chemotherapy in previously untreated patients with unresectable, locally advanced or metastatic urothelial carcinoma (DANUBE): A randomised, open-label, multicentre, phase 3 trial. Lancet Oncol..

[24] Motzer R.J., Escudier B., George S., Hammers H.J., Srinivas S., Tykodi S.S., Sosman J.A., Plimack E.R., Procopio G., McDermott D.F. (2020). Nivolumab versus everolimus in patients with advanced renal cell carcinoma: Updated results with long-term follow-up of the randomized, open-label, phase 3 CheckMate 025 trial. Cancer.

[25] Choueiri T.K., Powles T., Burotto M., Escudier B., Bourlon M.T., Zurawski B., Oyervides Juárez V.M., Hsieh J.J., Basso U., Shah A.Y. (2021). Nivolumab plus Cabozantinib versus Sunitinib for Advanced Renal-Cell Carcinoma. N. Engl. J. Med..

[26] Motzer R.J., Tannir N.M., McDermott D.F., Frontera O.A., Melichar B., Choueiri T.K., Plimack E.R., Barthélémy P., Porta C., George S. (2018). Nivolumab plus Ipilimumab versus Sunitinib in Advanced Renal-Cell Carcinoma. N. Engl. J. Med..

[27] Motzer R.J., Rini B.I., McDermott D.F., Aren Frontera O., Hammers H.J., Carducci M.A., Salman P., Escudier B., Beuselinck B., Amin A. (2019). Nivolumab plus ipilimumab versus sunitinib in first-line treatment for advanced renal cell carcinoma: Extended follow-up of efficacy and safety results from a randomised, controlled, phase 3 trial. Lancet Oncol..

[28] Choueiri T., Motzer R., Rini B., Haanen J., Campbell M., Venugopal B., Kollmannsberger C., Gravis-Mescam G., Uemura M., Lee J. (2020). Updated efficacy results from the JAVELIN Renal 101 trial: First-line avelumab plus axitinib versus sunitinib in patients with advanced renal cell carcinoma. Ann. Oncol..

[29] Powles T., Plimack E.R., Soulières D., Waddell T., Stus V., Gafanov R., Nosov D., Pouliot F., Melichar B., Vynnychenko I. (2020). Pembrolizumab plus axitinib versus sunitinib monotherapy as first-line treatment of advanced renal cell carcinoma (KEYNOTE-426): Extended follow-up from a randomised, open-label, phase 3 trial. Lancet Oncol..

[30] Common Terminology Criteria for Adverse Events (CTCAE)|Protocol Development|CTEP. https://ctep.cancer.gov/protocolDevelopment/electronic_applications/ctc.html.

[31] Abdel-Wahab N., Alshawa A., Suarez-Almazor M.E., Naing A., Hajjar J. (2017). Adverse Events in Cancer Immunotherapy. Immunotherapy. Advances in Experimental Medicine and Biology.

[32] Choueiri T.K., Fishman M.N., Escudier B., McDermott D.F., Drake C.G., Kluger H., Stadler W.M., Perez-Gracia J.L., McNeel D.G., Curti B. (2016). Immunomodulatory Activity of Nivolumab in Metastatic Renal Cell Carcinoma. Clin. Cancer Res..

[33] McDermott D.F., Lee J.-L., Bjarnason G.A., Larkin J.M.G., Gafanov R.A., Kochenderfer M.D., Jensen N.V., Donskov F., Malik J., Poprach A. (2021). Open-Label, Single-Arm Phase II Study of Pembrolizumab Monotherapy as First-Line Therapy in Patients with Advanced Clear Cell Renal Cell Carcinoma. J. Clin. Oncol..

[34] McDermott D.F., Sosman J.A., Sznol M., Massard C., Gordon M.S., Hamid O., Powderly J.D., Infante J.R., Fassò M., Wang Y.V. (2016). Atezolizumab, an Anti–Programmed Death-Ligand 1 Antibody, in Metastatic Renal Cell Carcinoma: Long-Term Safety, Clinical Activity, and Immune Correlates from a Phase Ia Study. J. Clin. Oncol..

[35] Vaishampayan U., Schöffski P., Ravaud A., Borel C., Peguero J., Chaves J., Morris J.C., Kotecki N., Smakal M., Zhou D. (2019). Avelumab monotherapy as first-line or second-line treatment in patients with metastatic renal cell carcinoma: Phase Ib results from the JAVELIN Solid Tumor trial. J. Immunother. Cancer.

[36] Postow M.A., Chesney J., Pavlick A.C., Robert C., Grossmann K., McDermott D., Linette G.P., Meyer N., Giguere J.K., Agarwala S.S. (2015). Nivolumab and Ipilimumab versus Ipilimumab in Untreated Melanoma. N. Engl. J. Med..

[37] Balar A.V., Castellano D., O’Donnell P.H., Grivas P., Vuky J., Powles T., Plimack E.R., Hahn N.M., de Wit R., Pang L. (2017). First-line pembrolizumab in cisplatin-ineligible patients with locally advanced and unresectable or metastatic urothelial cancer (KEYNOTE-052): A multicentre, single-arm, phase 2 study. Lancet Oncol..

[38] Programmed Cell Death 1 Forms Negative Costimulatory Microclusters That Directly Inhibit T Cell Receptor Signaling by Recruiting Phosphatase SHP2|Journal of Experimental Medicine|Rockefeller University Press. https://rupress.org/jem/article/209/6/1201/41299/Programmed-cell-death-1-forms-negative.

[39] Lidar M., Giat E., Garelick D., Horowitz Y., Amital H., Steinberg-Silman Y., Schachter J., Shapira-Frommer R., Markel G. (2018). Rheumatic manifestations among cancer patients treated with immune checkpoint inhibitors. Autoimmun. Rev..

[40] Kostine M., Rouxel L., Barnetche T., Veillon R., Martin F., Dutriaux C., Dousset L., Pham-Ledard A., Prey S., Beylot-Barry M. (2017). Rheumatic disorders associated with immune checkpoint inhibitors in patients with cancer—Clinical aspects and relationship with tumour response: A single-centre prospective cohort study. Ann. Rheum. Dis..

[41] Cappelli L.C., Gutierrez A.K., Baer A.N., Albayda J., Manno R.L., Haque U., Lipson E.J., Bleich K.B., Shah A.A., Naidoo J. (2016). Inflammatory arthritis and sicca syndrome induced by nivolumab and ipilimumab. Ann. Rheum. Dis..

[42] Calabrese C., Kirchner E., Kontzias K., Velcheti V., Calabrese L.H. (2017). Rheumatic immune-related adverse events of checkpoint therapy for cancer: Case series of a new nosological entity. RMD Open.

[43] Belkhir R., Le Burel S., Dunogeant L., Marabelle A., Hollebecque A., Besse B., Leary A., Voisin A.-L., Pontoizeau C., Coutte L. (2017). Rheumatoid arthritis and polymyalgia rheumatica occurring after immune checkpoint inhibitor treatment. Ann. Rheum. Dis..

[44] Calabrese C., Cappelli L.C., Kostine M., Kirchner E., Braaten T., Calabrese L. (2019). Polymyalgia rheumatica-like syndrome from checkpoint inhibitor therapy: Case series and systematic review of the literature. RMD Open.

[45] Yoshioka M., Kambe N., Yamamoto Y., Suehiro K., Matsue H. (2015). Case of respiratory discomfort due to myositis after administration of nivolumab. J. Dermatol..

[46] Hunter G., Voll C., Robinson C.A. (2009). Autoimmune Inflammatory Myopathy after Treatment with Ipilimumab. Can. J. Neurol. Sci./J. Can. Sci. Neurol..

[47] Barbosa N.S., Wetter D.A., Wieland C.N., Shenoy N., Markovic S.N., Thanarajasingam U. (2017). Scleroderma Induced by Pembrolizumab: A Case Series. Mayo Clin. Proc..

[48] Yamaguchi A., Saito Y., Okamoto K., Narumi K., Furugen A., Takekuma Y., Sugawara M., Kobayashi M. (2021). Preexisting autoimmune disease is a risk factor for immune-related adverse events: A meta-analysis. Support. Care Cancer.

[49] Danlos F.-X., Voisin A.-L., Dyevre V., Michot J.-M., Routier E., Taillade L., Champiat S., Aspeslagh S., Haroche J., Albiges L. (2018). Safety and efficacy of anti-programmed death 1 antibodies in patients with cancer and pre-existing autoimmune or inflammatory disease. Eur. J. Cancer.

[50] Alexander S., Swami U., Kaur A., Gao Y., Fatima M., Ginn M.M., Stein J.E., Grivas P., Zakharia Y., Singh N. (2021). Safety of immune checkpoint inhibitors in patients with cancer and pre-existing autoimmune disease. Ann. Transl. Med..

[51] Xie W., Huang H., Xiao S., Fan Y., Deng X., Zhang Z. (2020). Immune checkpoint inhibitors therapies in patients with cancer and preexisting autoimmune diseases: A meta-analysis of observational studies. Autoimmun. Rev..

[52] Tison A., Quéré G., Misery L., Funck-Brentano E., Danlos F., Routier E., Robert C., Loriot Y., Lambotte O., Bonniaud B. (2019). Safety and Efficacy of Immune Checkpoint Inhibitors in Patients with Cancer and Preexisting Autoimmune Disease: A Nationwide, Multicenter Cohort Study. Arthritis Rheumatol..

[53] Chanza N.M., Xie W., Issa M., Dzimitrowicz H., Tripathi A., Beuselinck B., Lam E., Zakharia Y., Mckay R., Shah S. (2019). Safety and efficacy of immune checkpoint inhibitors in advanced urological cancers with pre-existing autoimmune disorders: A retrospective international multicenter study. J. Immunother. Cancer.

[54] Cortellini A., Buti S., Santini D., Perrone F., Giusti R., Tiseo M., Bersanelli M., Michiara M., Grassadonia A., Brocco D. (2019). Clinical Outcomes of Patients with Advanced Cancer and Pre-Existing Autoimmune Diseases Treated with Anti-Programmed Death-1 Immunotherapy: A Real-World Transverse Study. Oncologist.

[55] Kim S.T., Tayar J., Trinh V.A., Suarez-Almazor M., Garcia S., Hwu P., Johnson D.H., Uemura M., Diab A. (2017). Successful treatment of arthritis induced by checkpoint inhibitors with tocilizumab: A case series. Ann. Rheum. Dis..

[56] Salem J.-E., Allenbach Y., Vozy A., Brechot N., Johnson D.B., Moslehi J.J., Kerneis M. (2019). Abatacept for Severe Immune Checkpoint Inhibitor–Associated Myocarditis. N. Engl. J. Med..

[57] Fountzilas E., Lampaki S., Koliou G.-A., Koumarianou A., Levva S., Vagionas A., Christopoulou A., Laloysis A., Psyrri A., Binas I. (2021). Real-world safety and efficacy data of immunotherapy in patients with cancer and autoimmune disease: The experience of the Hellenic Cooperative Oncology Group. Cancer Immunol. Immunother..

[58] National Cancer Institute (NCI) (2021). A Phase Ib Study of Nivolumab in Patients with Autoimmune Disorders and Advanced Malignancies (AIM-NIVO). https://clinicaltrials.gov/ct2/show/NCT03816345.

[59] Pantuck M., McDermott D., Drakaki A. (2019). To treat or not to treat: Patient exclusion in immune oncology clinical trials due to preexisting autoimmune disease. Cancer.

